# Spatiotemporal change in socioeconomic inequality in hygienic menstrual product use among adolescent girls in India during 2015–2019

**DOI:** 10.1186/s12939-023-02020-3

**Published:** 2023-09-29

**Authors:** Mahashweta Chakrabarty, Aditya Singh, Shivani Singh, Pooja Tripathi

**Affiliations:** 1 Department of Geography, Banaras Hindu University, Varanasi, Uttar Pradesh India; 2 GIRL Center, Population Council, NY USA; 3 Uttar Pradesh Technical Support Unit, India Health Action Trust, Lucknow, Uttar Pradesh India

**Keywords:** Health inequality, Economic inequality, Menstrual health and hygiene, Menstrual hygiene management, Hygienic products, Adolescent health, Erreygers’ concentration index, Socioeconomic inequality

## Abstract

**Background:**

The use of hygienic products, such as sanitary napkins, tampons, and menstrual cups, to absorb menstrual blood is vital for the health and well-being of adolescent girls in India. However, the degree of inequity in the use of such products among this subpopulation remains inadequately explored. To fill this critical knowledge gap, this study aims to investigate the spatiotemporal dynamics of hygienic product use among adolescent girls in India from 2015 to 2020.

**Methods:**

In this cross-sectional study, we analyzed data from 117,749 to 114,839 adolescent girls aged 15–19, obtained from two consecutive rounds of the National Family Health Survey (NFHS) conducted in India during 2015-16 and 2019-21. Our approach involved utilizing Erreygers’ Concentration Index (ECI) and Concentration Curve to quantitatively assess and visually represent socioeconomic inequality in hygienic product usage. Additionally, we investigated the spatiotemporal variation in this inequality over the study period and decomposed the ECI to identify the key contributing factors.

**Results:**

The findings reveal that hygienic product usage among adolescent girls in India has increased by 13 percentage points (PP), from 37% in 2015-16 to 50% in 2019-21. This increase is also visible across all household wealth quintiles. However, the bottom quintiles experienced a greater rise (+ 15 to 16 PP) than the top quintile (+ 8 PP). During the study period, the ECI reduced marginally, from 0.48 in 2015-16 to 0.43 in 2019-21. However, the extent of this reduction varied across different states. The greatest reduction in ECI was recorded in Punjab (-0.23 points), Telangana (-0.16 points), and West Bengal (-0.14 points). In contrast, there were a number of states with high socioeconomic inequality (ECI > 0.30) in 2015-16, where inequality reduction was minimal (< 0.05 points) over the study period. This included more developed states of Kerala, Karnataka, Maharashtra and Gujarat and relatively less developed states of Odisha, Jharkhand, Chhattisgarh, Uttar Pradesh, and Assam. Some states, such as Bihar and Madhya Pradesh, recorded an increase in socioeconomic inequality over the study period, with ECI rising to 0.31 and 0.46 (highest in the country) in 2019-21. The decomposition analysis revealed that the inequality in using hygienic products was primarily explained by place of residence, exposure to mass-media, education, and region of residence.

**Conclusions:**

The findings suggest the need for targeted policies to reduce existing socioeconomic inequality in the usage of hygienic products among adolescent girls in India. Specifically, interventions should target regions with low use of hygienic products, economically disadvantaged groups, and poor and vulnerable populations. State-specific policies and programs are also necessary to address the disparities in socioeconomic inequality. Additionally, efforts to reduce inequality should address the underlying factors contributing to inequality.

**Supplementary Information:**

The online version contains supplementary material available at 10.1186/s12939-023-02020-3.

## Introduction

Menstruation is a natural process experienced by girls and women from menarche to menopause [1]. With approximately 1.8 billion people menstruating every month and over 300 million girls and women menstruating at any given time, it is a widespread occurrence [2]. However, many societies still impose cultural taboos and discriminatory social norms on menstruation [3, 4]. Insufficient information about safe and hygienic menstruation further perpetuates unhygienic practices, misconceptions, and negative attitudes, leading to shaming, bullying, and gender-based violence [5, 6]. Consequently, poor menstrual health and hygiene exacerbate social and economic inequalities, significantly impacting girls’ and women’s education, health, safety, and overall development [7].

Adolescent girls are particularly vulnerable to poor menstrual hygiene management due to limited knowledge, experience, autonomy, and decision-making power [8, 9]. Inadequate access to comprehensive information and resources may lead to the adoption of unhygienic practices during menstruation, exposing them to significant health risks [10], including reproductive and urinary tract infections that can have profound implications for their overall well-being [11, 12]. Moreover, menstrual pain or heavy bleeding often contributes to school absenteeism and reduced social engagement, leading to potential social isolation and hindrances to their educational achievement [13–15]. The restricted access to comprehensive education and information related to sexuality, reproduction, and menstrual health, coupled with difficulties in obtaining menstrual products and healthcare services, create significant obstacles to effective menstruation management among adolescent girls [15]. This lack of knowledge, resources, or facilities can also give rise to feelings of embarrassment, discomfort, and social stigma [16]. Recognizing and addressing the importance of comprehensive menstrual hygiene contributes to their physical and emotional well-being and aligns with the overarching objectives of promoting health, education, and gender equality, as emphasized in the Sustainable Development Goals [17, 18].

In managing menstruation, girls and women utilize various menstrual hygiene products, including sanitary napkins, tampons, menstrual cups, and cloth (if properly managed) [19]. Research in low- and middle-income countries has highlighted that a significant proportion of women and girls resort to unhygienic materials during their menstrual periods due to the lack of affordable menstrual care products [15]. In the past two decades in India, various sanitary movements, awareness campaigns, and government and non-governmental initiatives and schemes to distribute subsidized sanitary napkins and other menstrual hygiene products have led to an increase in the use of hygienic products among women and girls in India [20, 21]. Government programs, while aiming to increase the overall usage of menstrual products among girls and women, must also prioritize inclusivity and equitable access. Progress towards universal usage should be guided by the principle of equity, with a focused effort to reduce existing socioeconomic and geographic disparities in the country. Every woman, regardless of her social or economic status, or place of residence, deserves equal opportunities to manage her menstruation with dignity and in a healthy manner. However, little is known about the extent to which the recent increase in the use of hygienic products among girls and women in India has been equitable or inequitable across wealth groups in different states of India.

Prior research in developing countries, including India, has consistently demonstrated a positive correlation between wealth and menstrual product use, revealing that economically disadvantaged women often encounter challenges accessing hygienic products, resulting in ‘period poverty’ [1, 9, 22–24]. These studies emphasize the necessity for interventions that address the economic barriers hindering women from accessing and affording menstrual products. However, there remains a dearth of research specifically focusing on adolescent girls in India and how socioeconomic inequality in hygienic product use has been evolving among this population. Understanding the trends and patterns of socioeconomic inequality in product use among adolescent girls is critical for addressing the persistent disparities they face in India. By gaining insights into these dynamics, targeted and evidence-based interventions can be devised to ensure equitable access to menstrual hygiene products, thus fostering the well-being and development of adolescent girls in the country.

This study, therefore, aims to examine the spatiotemporal change in the socioeconomic inequality in the use of hygienic products among adolescent girls in India from 2015 to 2019. Additionally, the paper aims to identify the contributing factors to socioeconomic inequality. The authors anticipate that the findings presented in this paper will provide insights for formulating targeted policies and strategies aimed at reducing disparities in hygienic product usage among adolescent girls in India. By addressing these disparities, this research endeavours to contribute to improved menstrual health and overall well-being for this vulnerable population.

## Data and methods

The data utilized in this study was obtained from two consecutive rounds of the National Family Health Survey (NFHS) conducted in India during 2015-16 and 2019-21. The NFHS covers various topics, including fertility, maternal and child health, nutrition, family planning, reproductive health, and domestic violence [25, 26]. The survey collects demographic and socioeconomic characteristics data at both the household and individual levels.

For this analysis, the sample was specifically restricted to adolescent girls aged 15–19. During the NFHS-4 conducted in 2015-16, a total of 699,686 women aged 15–49 were interviewed. From this group, 574,808 women above the age of 20 or outside the adolescent age range were excluded from the analysis. Subsequently, from the remaining 124,878 women aged 15–19, 7,129 were excluded due to missing information on age at menarche and social group. Ultimately, a final sample of 117,749 adolescent girls aged 15–19 from the NFHS-4 survey was considered for the study.

Similarly, in the more recent NFHS-5 conducted between 2019 and 2021, 724,115 adolescent girls aged 15–49 were interviewed. Among them, 601,635 women above 20 or outside the adolescent age range were excluded from the analysis. From the remaining 122,480 women aged 15–19, an additional 7,641 were excluded due to missing information on age at menarche and social group. Consequently, 114,839 adolescent girls aged 15–19 from the NFHS-5 survey were selected for inclusion in the final analysis.

### Statistical analysis

We utilized a combination of descriptive, bivariate, and multivariate analysis to understand the spatiotemporal change in the use of hygienic products among adolescent girls in India. First, we calculated the percentage of adolescent girls using hygienic products for India and its 28 states at two-time points, 2015-16 and 2019-21. These percentages were then graphically represented using bar graphs and dot plots, allowing for clear visualization of changes in hygienic product usage over time and across different states. Next, we measured and visualized the socioeconomic inequality in the use of hygienic products among adolescent girls by employing the Erreygers corrected concentration index (ECI) and concentration curve (CC). ECI provided us with a quantitative measure of the extent of socioeconomic inequality in hygienic products, while the CC visually represented this inequality. Subsequently, we conducted a regression-based decomposition analysis of the ECI to identify the key factors contributing to socioeconomic inequality in hygienic product usage. A detailed description of ECI, CC, and the decomposition of ECI is presented in the following sections.

### Corrected CI and CC

The concentration index (CI) is used to measure the extent to which the utilization of hygienic products is concentrated among specific socioeconomic groups, such as the wealthy or the poor. CI is calculated by plotting a CC, which shows the cumulative percentage of adolescent girls using hygienic products exclusively on the y-axis against the cumulative percentage of adolescent girls ranked by household wealth on the x-axis [27]. If the CI is negative and the CC is above the line of equality, the distribution of hygienic products use is concentrated among the poor. Conversely, if the CC is below the equality line and the CI is positive, the usage is concentrated among the rich [27]. A CI of zero indicates no socioeconomic inequality.

The standard CI is not well-suited for binary health variables because its bounds tend to shrink as the mean of the variable increases, leading to potential measurement issues. In response to this limitation, Wagstaff proposed a normalization method by dividing the index by the reciprocal of the variable’s mean or the CI’s bound [28]. However, Wagstaff’s index does not satisfy all four conditions of rank-dependent indices (i.e., mirror, transfer, level independence, and cardinal invariance). As a solution to address these shortcomings, Erreygers proposed a corrected concentration index, the ECI, to handle binary health variables, such as the usage of hygienic products in this study [29, 30]. The ECI is calculated using a formula that considers the socioeconomic rank of individuals and the maximum and minimum values of the health variable. By doing so, the ECI provides a more robust and reliable measure of socioeconomic inequality, allowing for a more accurate assessment of disparities in hygienic product usage among adolescent girls in India.

Erreygers has defined a corrected concentration index, $E\left(h\right)$, for binary variables as [30]:

$$E\left(h\right)=\frac{8\mu }{{n}^{2}({b}_{h}-{a}_{h)}}\sum _{i=1}^{n}{z}_{i}{h}_{i}$$

Where, where $${h}_{i}$$ is a binary variable that is equal to 1 if the adolescent girls use hygienic products exclusively and 0 otherwise; $${z}_{i}=\frac{(n+1)}{2}-{\lambda }_{i}$$ Where n is the sample size, and $${\lambda }_{i}$$ denotes the socioeconomic rank of the individual ranging from the richest ($${\lambda }_{i}$$ = 1) to the poorest ($${\lambda }_{i}$$ = n), $${b}_{h}$$ is the maximum value of the health variable and $${a}_{h)}$$ is the lowest value of health variable. The ECI also varies between − 1 and 1, similar to the standard CI.

### Decomposition analysis

Wagstaff’s decomposition method for CI, originally designed for continuous health variables, was not suitable for binary health variables like the usage of hygienic products [31, 32]. However, Erreygers addressed the technical limitations and introduced a corrected decomposition technique for the ECI. It involves calculating the sum of the product of the coefficients of independent variables and the generalized concentration index of each variable, along with the generalized concentration index of error terms (e*) [30]. It is represented by the following formula:

$$E\left(h\right)=4\left[\sum _{j=1}^{q}{\theta }_{j}^{*}V\left({x}_{j}\right)+V({e}^{*})\right]$$

Where ${\theta }_{j}^{*}$are the coefficients of independent variables, $V\left({x}_{j}\right)$ is the generalized concentration index of ${x}_{j}$ (independent variable) and $V\left({e}^{*}\right)$ is the generalized concentration index of error terms (${e}^{*}$).

For more information on the steps involved in the Erreygers decomposition, please see Erreygers (2009) [30].

### Variables

#### Dependent variable

During both NFHS-4 and NFHS-5 surveys, a multiple-response question was asked to gather information about the materials used by girls and women during their menstrual periods to absorb the blood and prevent blood stains. The question included six response options in NFHS-4: cloth, locally prepared napkins, sanitary napkins, tampons, others, and nothing. In NFHS-5, an additional option for menstrual cup usage was introduced, making it a total of seven response options. Based on these responses, a binary outcome variable called “use of hygienic products” was created. Specifically, girls and women who reported using only sanitary napkins, locally made napkins, or tampons were coded as “1”, indicating the use of hygienic products. In contrast, those who reported using non-hygienic products such as cloth, those who did not use any menstrual products at all, or those who used both hygienic and non-hygienic products simultaneously were coded as “0”, indicating the use of non-hygienic products. In the absence of specific information on washing and drying practices for cloth usage during menstruation in the NFHS dataset, it is not possible to determine if the cloth is being used in a hygienic manner. To maintain consistency and avoid assumptions, the study has classified cloth as a non-hygienic material in the analysis. Additionally, as menstrual cup usage was not included as an option in NFHS-4, it was not considered in defining the dependent variable for this study.

### Independent variables

As the NFHS surveys did not provide direct data on income and expenditure, the study utilized the household wealth index as a reliable proxy for assessing the economic status of each household. The computation of the ‘wealth index’ was based on principal component analysis (PCA), which considered various indicators, such as housing quality, household amenities, consumer durables, and land holding size. Each household was assigned a ‘wealth score’ based on the presence or absence of these indicators and subsequently ranked based on the wealth score and categorized into five distinct ‘wealth quintiles’: poorest, poorer, middle, richer, and richest [33]. Given that the wealth score and wealth index variables were pre-computed as part of the NFHS surveys, the authors did not have to create these variables separately. Instead, they directly utilized the existing variables in their analysis.

This study incorporated relevant independent variables based on existing literature on menstrual health and hygiene [11, 22, 34–37]. The selected variables included the age at menarche (in years), marital status (currently not married, currently married), level of education of the respondent (below secondary, secondary and above), religion (Hindu/non-Hindu), social groups (Scheduled Caste/Scheduled Tribe or SC/ST, Non-SC/ST), mass media exposure (no mass media exposure, exposed to at least one medium), and region of residence (northern, central, western, eastern, southern, northeastern), and place of residence (rural, urban). These variables were selected to identify their potential contributions to the existing socioeconomic inequality observed in the use of hygienic products during menstruation among adolescent girls, as these variables are theorized to be associated with the existing inequality, specifically in menstrual product use.

## Results

### Sample characteristics

The study sample consisted of 117,749 adolescent girls from NFHS-4 and 114,839 from NFHS-5. The majority of girls in both rounds experienced their first period between the ages of 13 and 15 (see Table 1). Around 80% of the girls had received education up to the secondary level, with most of them identifying as Hindu. Less than half of the girls belonged to the Other Backward Classes (OBCs), while approximately 15–20% reported no mass media exposure. A large majority (85–88%) of the girls were not currently married, and nearly 30% of the sample came from the central region of the country.

**Table 1:** Descriptive statistics of adolescent girls in India, NFHS 4 and NFHS 5

Background Characteristics	NFHS-4		NFHS-5	
	N (117, 749)	%	N (114, 839)	%
Age at menarche (in years)				
≤ 12	22,400	19.02	20,305	17.68
13–15	92,332	78.41	91,604	79.77
≥ 16	3,016	2.56	2,930	2.55
**Education**				
No education	8,057	6.84	5,063	4.41
Primary	8,193	6.96	5,948	5.18
Secondary	93,376	79.30	95,215	82.91
Higher	8,123	6.90	8,613	7.50
**Religion**				
Hindu	94,796	80.51	94,277	82.09
Muslim	17,229	14.63	15,548	13.54
Christian	2,317	1.97	2,323	2.02
Others	3,406	2.89	2,692	2.34
**Social groups**				
SC	26,208	22.26	27,895	24.29
ST	11,671	9.91	11,530	10.04
OBC	54,641	46.40	53,211	46.34
Other	25,229	21.43	22,203	19.33
**Exposure to mass media**				
No exposure to mass media	18,070	15.35	22,115	19.26
At least exposed to any one kind of mas	99,679	84.65	92,724	80.74
**Marital status**				
Currently not married	1,00,108	85.02	1,00,944	87.90
Currently married	17,641	14.98	13,895	12.10
**Region of residence**				
Northern	15,540	13.20	16,507	14.37
Central	34,900	29.64	35,040	30.51
Eastern	27,413	23.28	27,771	24.18
Western	15,546	13.20	13,658	11.89
Southern	20,993	17.83	19,061	16.60
Northeastern	3,357	2.85	2,803	2.44
**Household wealth**				
Poorest	24,389	20.71	25,190	21.94
Poorer	26,344	22.37	26,072	22.70
Middle	25,124	21.34	24,232	21.10
Richer	22,874	19.43	21,617	18.82
Richest	19,018	16.15	17,727	15.44
**Place of residence**				
Urban	35,719	30.34	32,269	28.10
Rural	82,030	69.66	82,570	71.90

Note: N = sample size; CI = confidence interval; SC = Scheduled Caste; ST = Scheduled Tribe; OBC = Other Backward Classes; all percentages are weighted

#### Use of hygienic products by background characteristics

The study reveals an upward trend in the use of hygienic products during menstruation among adolescent girls in India, with an increase of 13% points, from 37% in 2015-16 (NFHS-4) to 50% in 2019–2021 (NFHS-5). The data indicate that the use of hygienic products is positively associated with the level of education, as girls with higher education demonstrated higher usage rates in both rounds of NFHS. Notably, girls without formal education exhibited a significant increase in usage, doubling from 8% in NFHS-4 to 18% in NFHS-5 (refer to Table 2). The analysis revealed significant variations in hygienic product usage among different religions and social groups in the country, with the highest usage reported among adolescent girls from the general category (61.4%) and the lowest reported among ST girls, standing at 25% in NFHS-4 and 40% in NFHS-5. Furthermore, there was a substantial gap in usage between girls with no mass media exposure and those with any form of media exposure across both NFHS rounds.

**Table 2:** Percentage of adolescent girls using hygienic products by background characteristics in India, NFHS-4 (2015-16) and NFHS-5 (2019-21)

Background characteristics	NFHS-4 (2015-16)		NFHS-5 (2019-21)	
	% of girls using hygienic products N = 117,749	95% CI [Lower, Upper]	% of girls using hygienic products N = 114,839	95% CI [Lower, Upper]
Age at menarche (in years)				
≤ 12	38.70	[37.51, 39.90]	53.74	[52.56, 54.92]
13–15	36.24	[35.55, 36.93]	49.16	[48.52, 49.81]
≥ 16	38.41	[35.81, 41.08]	54.61	[52.18, 57.03]
**Education**				
No education	8.14	[7.318, 09.05]	17.56	[16.10, 19.14]
Primary	12.86	[11.76, 14.06]	22.46	[20.98, 24.02]
Secondary	39.15	[38.48, 39.83]	51.85	[51.22, 52.47]
Higher	61.77	[60.06, 63.46]	69.17	[67.67, 70.62]
**Religion**				
Hindu	37.18	[36.49, 37.87]	50.35	[49.70, 51.00]
Muslim	30.14	[28.62, 31.70]	43.35	[41.75, 44.97]
Christian	54.87	[51.63, 58.07]	63.51	[60.66, 66.26]
Others	46.39	[43.16, 49.64]	69.33	[67.00, 71.57]
**Social groups**				
SC	34.84	[33.67, 36.04]	48.94	[47.86, 50.01]
ST	24.73	[23.42, 26.08]	39.96	[38.58, 41.34]
OBC	35.69	[34.84, 36.54]	48.21	[47.41, 49.01]
Other	46.65	[45.37, 47.93]	61.42	[60.16, 62.66]
**Exposure to mass media**				
No exposure to mass media	9.67	[9.026, 10.36]	27.23	[26.30, 28.18]
At least exposed to any one kind of mass media	41.67	[40.99, 42.36]	55.57	[54.93, 56.20]
**Marital status**				
Currently not married	38.15	[37.47, 38.82]	50.59	[49.95, 51.22]
Currently married	28.91	[27.75, 30.10]	46.64	[45.34, 47.95]
**Region of residence**				
Northern	45.16	[43.81, 46.51]	65.78	[64.62, 66.92]
Central	18.40	[17.72, 19.09]	30.48	[29.62, 31.37]
Eastern	24.86	[23.82, 25.92]	44.17	[42.95, 45.41]
Western	50.85	[48.74, 52.96]	64.64	[62.79, 66.44]
Southern	67.65	[66.21, 69.06]	72.50	[71.16, 73.81]
Northeastern	27.65	[26.08, 29.27]	38.94	[37.24, 40.66]
**Place of residence**				
Urban	57.87	[56.58, 59.15]	68.76	[67.54, 69.95]
Rural	27.57	[26.95, 28.19]	42.82	[42.19, 43.46]

Note: N = sample size; CI = confidence interval; SC = Scheduled Caste; ST = Scheduled Tribe; OBC = Other Backward Classes; all percentages are weighted

Upon further analysis of the study results, it becomes evident that there is a significant regional variation in the use of hygienic products among adolescent girls in India. In 2015-16, the southern region reported the highest usage rate at 67.7%, while the central region reported the lowest usage rate at 18.4%. Despite an overall increase in usage across all regions during the study period, a notable regional disparity in the use of hygienic products persists. India’s northern and eastern regions experienced a substantial increase of over 20% points in the use of hygienic products, indicating positive progress. However, in the central and northeastern regions, where the initial usage was relatively low (18%), the increase was comparatively modest at around 11 to 12% points during the study period. The southern region, which already had a high prevalence of hygienic product usage (68%) during NFHS-4, saw only a marginal increase of 5% points during the study period.

**Fig. 1:**
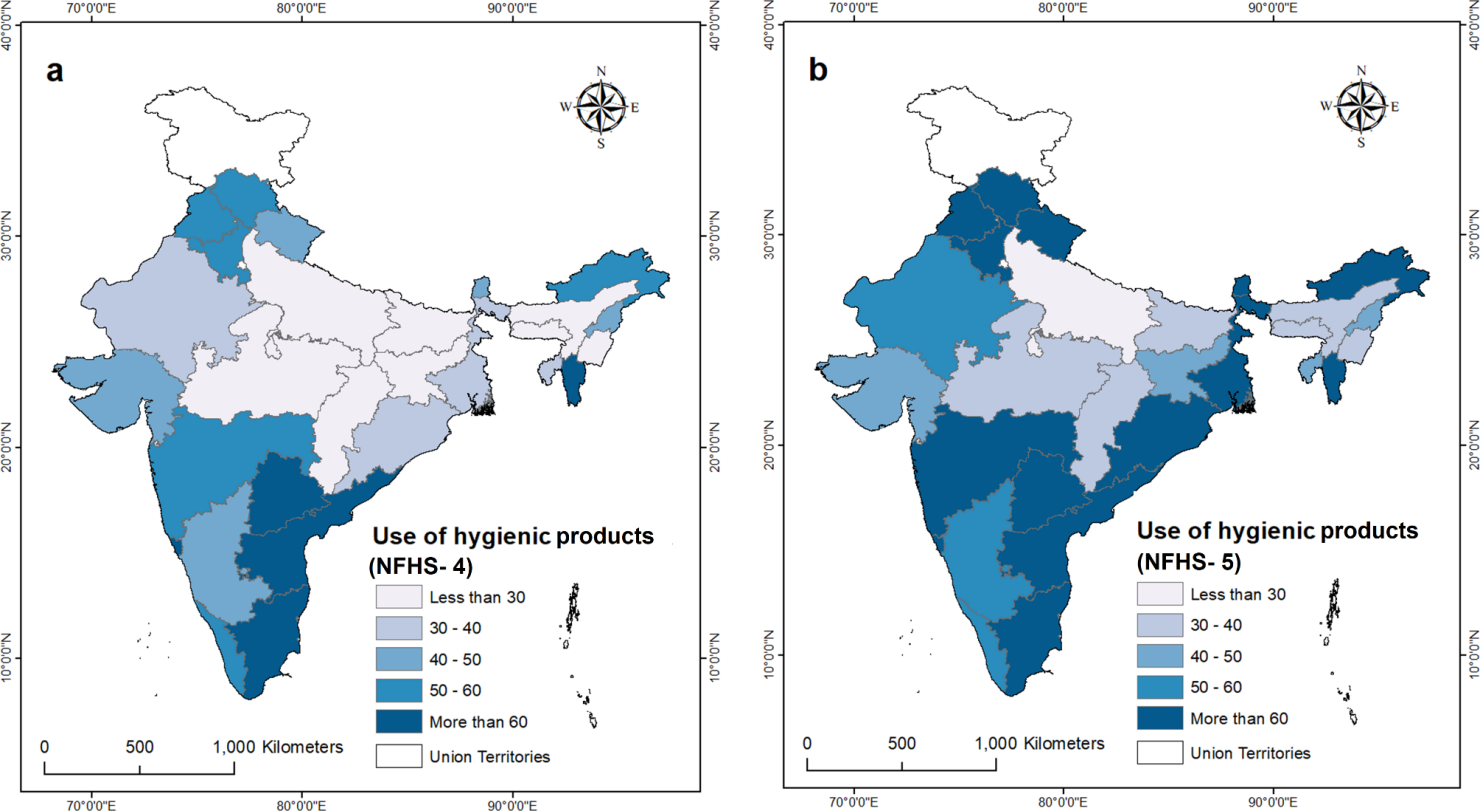
State-wise use of hygienic products among adolescent girls, (a) NFHS-4 (2015-16), and (b) NFHS-5 (2019-21)

Figure 1 unravel the state-level variation in using hygienic products among adolescent girls during the study period. The change in the usage of hygienic products between NFHS-4 and NFHS-5 varied across states. Among the 28 states, 25 experienced a positive change in usage. Goa, Gujarat, and Mizoram observed a slight decline in usage during the study period. The change in usage ranged between 20 and 30% points in Odisha, Rajasthan, West Bengal, Sikkim, Haryana, Punjab, and Maharashtra. On the other hand, six states, including Tamil Nadu, Andhra Pradesh, Kerala, Manipur, Meghalaya, and Nagaland, witnessed a change in usage of less than 10% points (see Appendix Table 1).

#### Socioeconomic inequality in the use of hygienic products among adolescent girls

**Fig. 2:**
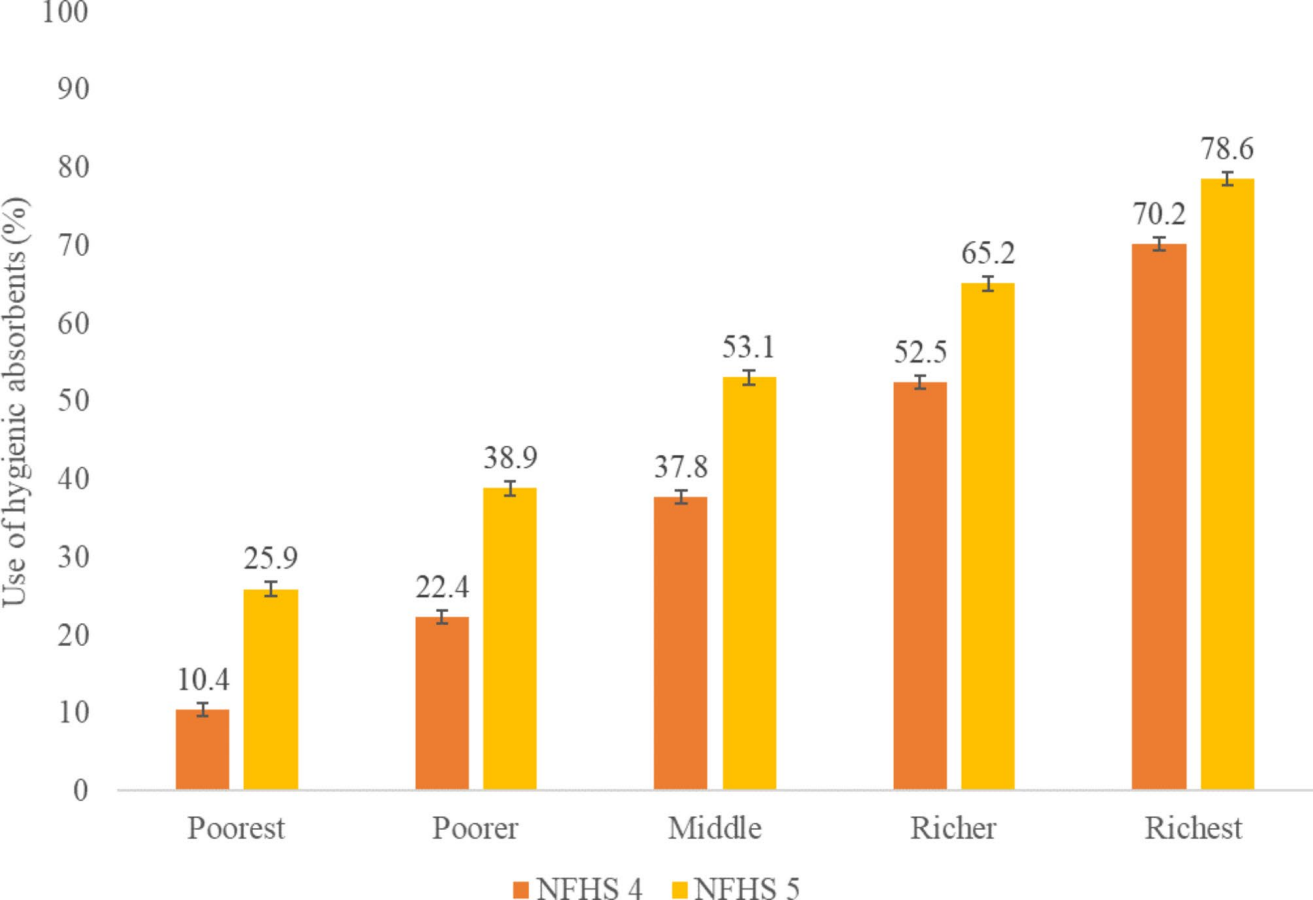
Use of hygienic products by household wealth quintiles among adolescent girls in India, NFHS-4 (2015-16) and NFHS-5 (2019-21)

The study shows that the usage of hygienic products among adolescent girls in India has increased as household wealth increases, as observed in both rounds of NFHS. However, this increase is not uniform across all wealth quintiles. Notably, in the two lowest wealth quintiles, there was a significant rise of 16–17% points in hygienic product usage during the study period. For instance, in the poorest quintile, the usage increased from 10% (95% CI: 9.8–11.0) to 26% (95% CI: 25.1–26.8), and in the poorer quintile, it increased from 22% (95% CI: 21.5–23.3) to 39% (95% CI: 38.0-39.8). On the other hand, the increase in hygienic product usage was relatively smaller in the richest wealth quintile, with only a 9% point rise, from 70% (95% CI: 68.9–71.4) in 2015-16 to 79% (95% CI: 77.5–79.6) in 2019-21 (see Fig. 2).

#### Spatiotemporal variation in socioeconomic inequality

**Fig. 3:**
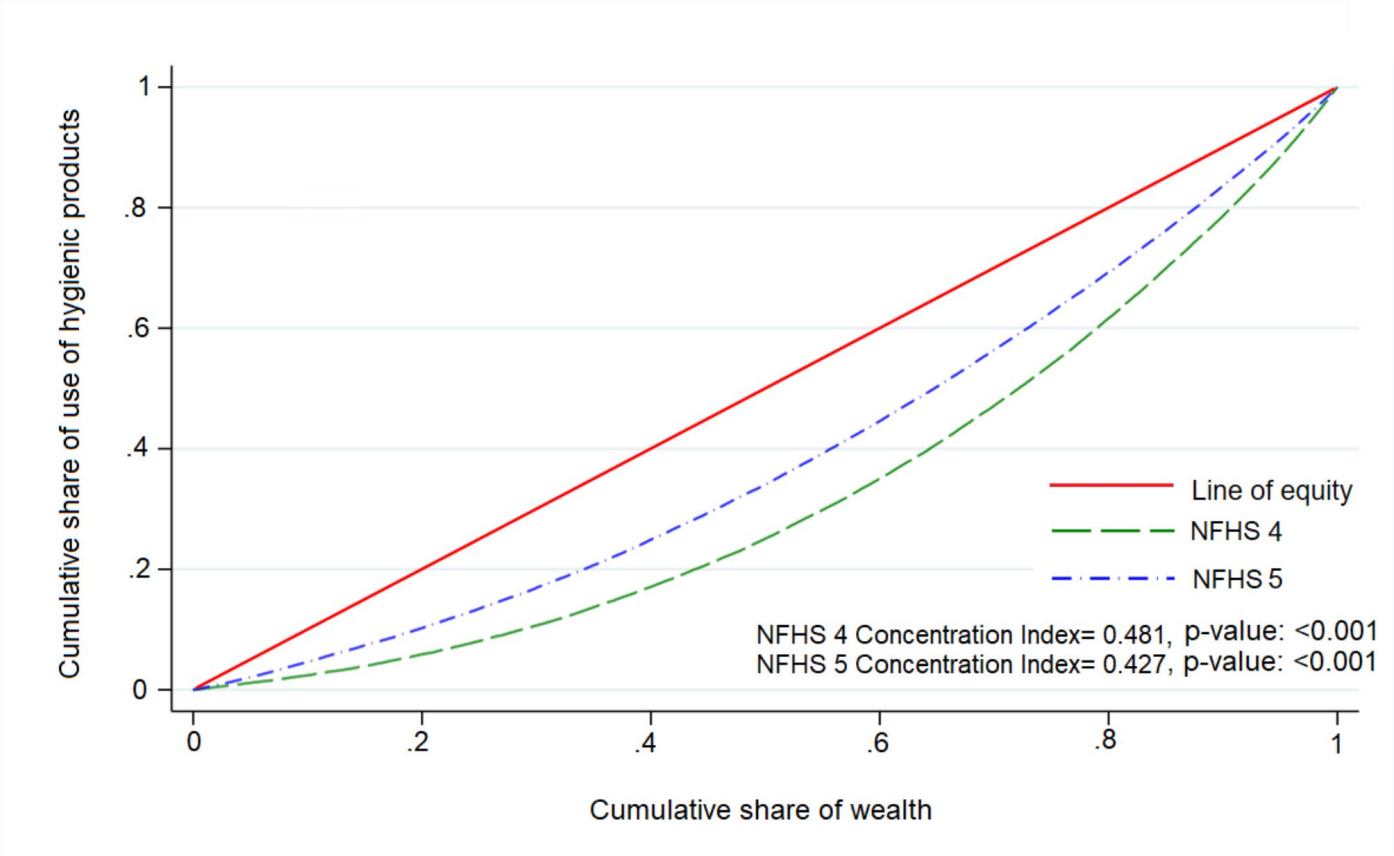
Concentration curve showing socioeconomic inequalities in the use of hygienic products among adolescent girls in India, NFHS-4 (2015-16) and NFHS-5 (2019-21)

The ECI for the country decreased from 0.48 in 2015-16 to 0.43 in 2019-21, indicating a marginal decline in socioeconomic inequality in the use of hygienic products among adolescent girls in India during the study period. It is also visible in CC, shown in Fig. 3.

Despite reducing the magnitude of socioeconomic inequality in the use of hygienic products between NFHS-4 and NFHS-5, a significant pro-rich inequality persists [38, 39]. Moreover, the extent of this inequality varies considerably across different states in India (see Table 3). In NFHS-5, the highest inequality was observed in Madhya Pradesh (ECI = 0.46, p < 0.001), while Tamil Nadu reported the lowest inequality (ECI = 0.10, p < 0.001). The ECIs ranged from 0.30 to 0.40 for eleven states, including the seven Empowered Action Group states of Assam, Bihar, Jharkhand, Odisha, Uttar Pradesh, Rajasthan, and Uttarakhand.

**Table 3:** Socioeconomic inequality in the use of hygienic products among adolescent girls across the states of India during NFHS-4 (2015-16) and NFHS-5 (2019-21)

States/ UTs	NFHS-4 (2015-16)			NFHS-5 (2019-21)			Change during 2015–2021		
	ECI	SE	p-value	ECI	SE	p-value	Difference	SE	p-value
Tamil Nadu	0.112	0.013	< 0.001	0.101	0.013	< 0.001	-0.010	0.018	0.562
Telangana	0.280	0.031	< 0.001	0.122	0.015	< 0.001	-0.158	0.034	***< 0.001***
Goa	0.289	0.072	< 0.001	0.157	0.080	0.052	-0.131	0.108	0.222
Sikkim	0.216	0.039	< 0.001	0.198	0.049	< 0.001	-0.018	0.063	0.773
Haryana	0.325	0.019	< 0.001	0.201	0.016	< 0.001	-0.124	0.025	***< 0.001***
Punjab	0.446	0.021	< 0.001	0.221	0.018	< 0.001	-0.225	0.027	***< 0.001***
Andhra Pradesh	0.308	0.029	< 0.001	0.226	0.028	< 0.001	-0.082	0.040	***0.043***
Himachal Pradesh	0.280	0.030	< 0.001	0.234	0.028	< 0.001	-0.045	0.042	0.275
Mizoram	0.190	0.016	< 0.001	0.258	0.025	< 0.001	0.069	0.030	***0.020***
Manipur	0.254	0.023	< 0.001	0.267	0.033	< 0.001	0.013	0.040	0.746
Arunachal Pradesh	0.397	0.025	< 0.001	0.274	0.019	< 0.001	-0.123	0.031	***< 0.001***
Kerala	0.299	0.030	< 0.001	0.277	0.029	< 0.001	-0.021	0.042	0.611
Tripura	0.413	0.040	< 0.001	0.286	0.037	< 0.001	-0.126	0.055	***0.020***
Chhattisgarh	0.324	0.013	< 0.001	0.289	0.015	< 0.001	-0.035	0.020	0.080
West Bengal	0.438	0.020	< 0.001	0.296	0.021	< 0.001	-0.142	0.029	***< 0.001***
Maharashtra	0.341	0.016	< 0.001	0.303	0.014	< 0.001	-0.038	0.022	0.077
Bihar	0.249	0.008	< 0.001	0.306	0.010	< 0.001	0.057	0.013	***< 0.001***
Assam	0.315	0.015	< 0.001	0.307	0.018	< 0.001	-0.008	0.023	0.719
Karnataka	0.336	0.019	< 0.001	0.313	0.017	< 0.001	-0.022	0.025	0.379
Gujarat	0.313	0.018	< 0.001	0.314	0.016	< 0.001	0.000	0.024	0.984
Uttar Pradesh	0.310	0.006	< 0.001	0.319	0.007	< 0.001	0.009	0.009	0.335
Uttarakhand	0.417	0.019	< 0.001	0.338	0.023	< 0.001	-0.078	0.030	***0.008***
Jharkhand	0.377	0.013	< 0.001	0.343	0.016	< 0.001	-0.035	0.020	0.087
Odisha	0.382	0.014	< 0.001	0.347	0.017	< 0.001	-0.035	0.022	0.108
Rajasthan	0.448	0.011	< 0.001	0.355	0.012	< 0.001	-0.094	0.016	***< 0.001***
Nagaland	0.409	0.026	< 0.001	0.376	0.030	< 0.001	-0.033	0.040	0.410
Meghalaya	0.362	0.023	< 0.001	0.409	0.022	< 0.001	0.047	0.032	0.135
Madhya Pradesh	0.388	0.008	< 0.001	0.456	0.011	< 0.001	0.068	0.014	***< 0.001***

Note: ECI = Erreygers concentration index, SE = standard error

**Fig. 4:**
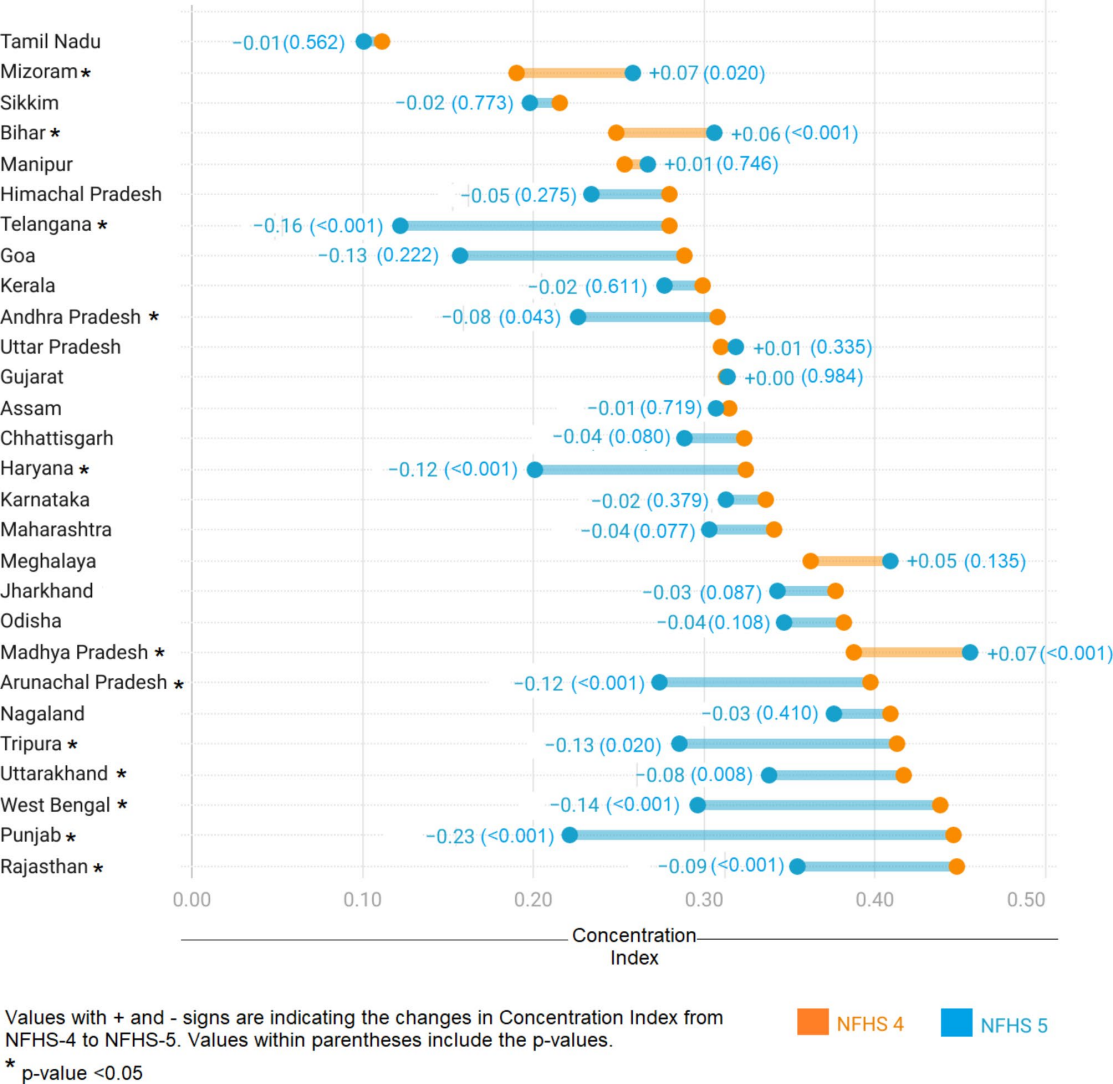
Change in socioeconomic inequality in the use of hygienic products among adolescent girls in India during NFHS-4 (2015-16) to NFHS-5 (2019-21)

In terms of changes in inequality, out of 28 states, only nine (Andhra Pradesh, Arunachal Pradesh, Haryana, Punjab, Rajasthan, Telangana, Tripura, West Bengal, and Uttarakhand) showed a statistically significant reduction in inequality, as illustrated in Fig. 4. The greatest reduction in ECI was recorded in Punjab (-0.23 points), Telangana (-0.16 points), and West Bengal (-0.14 points). In contrast, there were a number of states with high socioeconomic inequality (ECI > 0.30) in 2015-16, where inequality reduction was minimal (< 0.05 points) or negligible over the study period. This included both more developed states of Kerala, Karnataka, Maharashtra and Gujarat and relatively less developed states of Odisha, Jharkhand, Chhattisgarh, Uttar Pradesh, and Assam. Some states, such as Bihar and Madhya Pradesh, recorded an increase in socioeconomic inequality over the study period, with ECI rising over 0.31 and 0.46 (highest in the country) in 2019-21.

#### Decomposition of the socioeconomic inequality in the use of hygienic products

The decomposition analysis reveals the main contributors to inequality in the exclusive use of hygienic products among adolescent girls. Table 4 presents the result of the decomposition analysis. Our study identified that place of residence (42%) is the main contributing factor to socioeconomic inequality in the use of hygienic products. Other 45% of the socioeconomic inequalities were explained by two variables, i.e., mass-media exposure (24%) and education (21%).

**Table 4:** Decomposition of socioeconomic inequality in the use of hygienic products among adolescent in India

Variable	Elasticity	CI	Contribution	Contribution (%)
**Age at menarche** (≥ 13 years)	0.024	-0.015	0.000	-0.06
**Education** (secondary and above)	0.748	0.169	0.126	21.74
**Religion** (Hindu)	0.119	-0.072	-0.009	-1.47
**SC/ST**	-0.039	-0.238	0.009	1.58
**Mass media exposure** (At least exposed to any one kind of mass media)	0.446	0.318	0.142	24.42
**Marital status** (currently married)	-0.001	-0.080	0.000	0.01
**Place of residence** (rural)	-0.518	-0.475	0.246	42.31
**Region of residence**				
*Northern*	0.083	0.143	0.012	2.05
*Central*	-0.125	-0.127	0.016	2.75
*Eastern*	0.013	-0.271	-0.004	-0.61
*Western*	0.069	0.099	0.007	1.18
*Southern*	0.165	0.177	0.029	5.04
*Northeastern*	0.000	-0.022	0.000	0.00
**Year**	0.277	0.022	0.006	1.07
Explained ECI		0.370		100
Actual ECI		0.402		
Unexplained ECI		0.032		

Note: ECI = Concentration index; SC/ST: Scheduled Caste/Scheduled Tribe

## Discussion

The study aimed to measure and analyze the spatiotemporal change in socioeconomic inequality in the use of hygienic products among adolescent girls in India between 2015-16 and 2019-21. To achieve this objective, the study used data from two rounds of the NFHS conducted in 2015-16 and 2019-21. The study reveals that the use of hygienic products among adolescent girls in India has increased from 37% in 2015-16 to 50% in 2019-21, yet significant regional variation in usage persists. Specifically, the states within northern and eastern regions demonstrated more increases in the use of hygienic products compared to the southern states, where the use had already been high in the previous round. This finding highlights the importance of recognizing state-wise disparities in the usage of hygienic products and tailoring interventions accordingly. While the southern states have made considerable progress, focusing on sustaining and further promoting the existing high usage levels, the central, eastern, and northeastern states require targeted interventions to accelerate the adoption of hygienic products.

The findings of the study reaffirmed the positive relationship between the use of hygienic products and household wealth, as previously observed in other studies [23, 38, 40]. Evidently, there was a progressive increase in product usage as one moved from the poorest to the richer quintiles. However, what stood out during the study period was that adolescent girls from the poorer and poorest quintiles experienced a more significant increase in the use of hygienic products during menstruation compared to the other quintiles. This positive trend contributed to a slight decline in socioeconomic inequality over the study period [38, 41]. However, findings also revealed that despite this overall progress, the reduction in socioeconomic inequality in hygienic product usage among adolescent girls was considerably heterogeneous across different states in India. Some states, such as Punjab, Telangana, and West Bengal, demonstrated commendable efforts in narrowing the inequality gap during the study period. However, a significant number of states did not experience substantial progress in reducing inequality or, in some cases, even witnessed an increase in inequality, suggesting the need for more concerted efforts to reduce socioeconomic inequality in the use of hygienic products among adolescent girls in these states. Interestingly, this pattern was observed in both more developed states like Kerala, Karnataka, Maharashtra, and Gujarat and relatively less developed states such as Odisha, Jharkhand, Chhattisgarh, Bihar, Madhya Pradesh, Uttar Pradesh, and Assam. The presence of this pattern in states with varying levels of development underscores the complexity of the challenges involved.

The study employed a decomposition analysis to gain insights into the factors influencing the existing inequality in the use of hygienic products. The results indicated that rural residence, exposure to mass media, and education were the primary drivers of this inequality. These findings underscore the importance of directing policies and interventions towards addressing the needs of rural populations, especially focusing on poor and uneducated rural girls. Additionally, promoting mass media campaigns to increase awareness and access to information can play a vital role in reducing the disparities in hygienic product usage among adolescent girls [41]. In rural areas of India, challenges such as inadequate sanitation facilities, limited access to healthcare services, and poor infrastructure contribute to suboptimal menstrual hygiene management practices and restricted availability of hygienic products, especially among economically disadvantaged groups [42–44]. Moreover, rural girls typically have lower levels of education [45] and are often engaged in agricultural or manual labour, which can further impede their ability to afford and obtain menstrual products [46].

Previous studies have emphasized the significance of education in enhancing menstrual hygiene for women and girls [14, 41]. Limited education can lead to a lack of awareness and knowledge, hindering the ability to address cultural barriers and seek assistance for menstrual health concerns [47, 48]. It can also amplify financial constraints, making accessing and affording hygienic products difficult. Furthermore, limited education can restrict economic opportunities, further complicating the affordability of menstrual products [22]. Mass media exposure is crucial in promoting awareness of menstrual hygiene management and challenging cultural taboos and myths associated with menstruation, which may disproportionately affect poor girls and women [49, 50]. It serves as a platform for disseminating information about the availability, affordability, and proper usage of menstrual products, facilitating easier access and utilization by adolescent girls from low-income backgrounds [16, 50]. Notably, advertisements highlighting the advantages of hygienic products, such as comfort and convenience, can encourage adolescent girls to transition from unhygienic alternatives [51].

The Government of India has undertaken initiatives to cater the needs of economically disadvantaged adolescent girls and women, particularly through the Scheme for Promotion of Menstrual Hygiene implemented by the Ministry of Health and Family Welfare (MOHFW) in 2011 [52]. This program focuses on adolescent girls aged 10–19 years and offers subsidized sanitary napkins called ‘Free Days’ at a nominal cost of Rs. 1 per napkin, distributed through accredited social health activists (ASHAs), who serve as frontline health workers [53]. Despite these efforts, challenges pertaining to procurement, supply, high costs, and limited enthusiasm among ASHAs have hindered the effective implementation of this initiative [54, 55]. In 2018, the Central Government introduced ‘*Suvidha*,‘ a 100% biodegradable sanitary napkin available at subsidized prices through government-run pharmacies known as *Jan Aushadhi Kendras* [21]. However, the limited number of pharmacies, primarily concentrated in metropolitan areas, has left a significant population underserved [56]. The scheme’s performance has not met its goals in many states, necessitating a comprehensive evaluation to improve its effectiveness and impact on adolescent girls’ menstrual hygiene in India [56].

Moreover, various state governments across the country have implemented programs to improve access to hygienic products, particularly sanitary pads, for adolescent girls [9]. However, the effectiveness of these programs has varied across different states. Notably, Tamil Nadu’s “*Pudhu Yugam*” scheme has demonstrated commendable outcomes by providing free monthly sanitary napkins to women and girls in the state [5, 38]. This has resulted in higher usage rates and minimal socioeconomic disparities [5]. In contrast, states like Telangana have encountered challenges in addressing socioeconomic inequality despite achieving high usage rates through similar programs [57]. In states such as Uttar Pradesh (*Kishori Suraksha Yojna*), Madhya Pradesh (*Udita Yojana*), and Bihar (*Kishori Shakti Yojana*), the implementation of such schemes has yielded neither substantial improvements in usage rates nor significant reductions in socioeconomic inequality regarding the utilization of hygienic products among adolescent girls in these states [58–60]. It is crucial to recognize that previous studies have shed light on various challenges, including corruption, supply-side issues, and difficulties in distribution and affordability [55, 61, 62]. These factors have likely impeded the effective implementation of interventions targeted at reaching economically disadvantaged adolescent girls.

Significant socioeconomic inequality in the usage of hygienic products and the uneven progress in reducing this disparity among different states, as revealed by this study, underscores the critical importance of prioritizing equity and spatial justice in policies and programs related to menstrual hygiene [63]. Merely focusing on increasing usage rates at the state or national level without addressing disparities among socioeconomic groups is unfair and unjust [64]. Therefore, to ensure equity and spatial justice, policies and programs concerning menstrual hygiene must extend beyond broad targets and consider the unique needs and circumstances of economically disadvantaged group of adolescent girls, especially in areas with limited access to hygienic products.

Several limitations to this study should be acknowledged. Firstly, the study relied on self-reported data from the NFHS, which could be subject to response bias and inaccuracies in reporting. Secondly, cloth which is a widely used menstrual hygiene material, can be categorized as both hygienic and unhygienic depending on the way it is washed, dried and sanitized, has been categorized clothes as unhygienic material in this study because the NFHS data does not provide any information about the washing and drying practices. This limitation of our outcome variable should be considered while interpreting the results. Finally, the study did not consider the impact of cultural norms and taboos surrounding menstruation on the use of hygienic products, which could also contribute to inequality in menstrual product use among adolescent girls. Despite these limitations, this study provides valuable insights into the spatiotemporal changes in socioeconomic inequality in menstrual hygiene product use among adolescent girls in India. It underscores the urgent need for targeted policies and programs to address the persistent disparities.

## Conclusion

In conclusion, this study reveals important insights into the spatiotemporal dynamics of socioeconomic inequality in the use of hygienic products among adolescent girls in India from 2015-16 to 2019-21. Reducing pro-rich inequality in hygienic product usage signifies positive steps towards promoting equitable access to menstrual hygiene products among adolescent girls in India. However, it is crucial to acknowledge that challenges persist in certain states, and the reduction in inequality is not uniform across the country. Some states showed commendable progress in reducing inequality, while others witnessed either an increase, minimal, or no change. Interestingly, this pattern was observed in more and relatively less developed states. This highlights the heterogeneity in the challenges and opportunities different states face in promoting menstrual health and hygiene among adolescent girls. The decomposition analysis highlighted the key factors influencing inequality, with place of residence, exposure to mass media, education, and region playing pivotal roles. These findings underscore the need for targeted interventions, particularly in rural areas and among poor, uneducated rural girls, to improve access to information through mass media campaigns and address infrastructure and affordability challenges. Overall, this study provides valuable insights for policymakers, advocates, and stakeholders working towards enhancing menstrual hygiene and promoting a more inclusive and equal society for adolescent girls in India. By recognizing and addressing disparities, we can ensure that all girls have the opportunity to manage their menstruation with dignity and without hindrance to their education and overall well-being.

### Electronic supplementary material

Below is the link to the electronic supplementary material.

Supplementary Material 1

## Data Availability

The National Family Health Survey-5 dataset used in this study is publicly available on the official website of Demographic and Health Surveys (DHS) at https://dhsprogram.com/data/availabledatasets.cfm. Researchers and data users can access this dataset by registering as a DHS data user and requesting access for legitimate research purposes through the following link: https://dhsprogram.com/data/Access-Instructions.cfm.
