# Supplementary material for: Spatiotemporal change in socioeconomic inequality in hygienic menstrual product use among adolescent girls in India during 2015–2019
Source: Int J Equity Health. 2023 Sep 29;22:202. doi: 10.1186/s12939-023-02020-3 (PMC10543847; doi:10.1186/s12939-023-02020-3)
Supplement: Supplementary file 1 — Supplementary Material 1 [file 12939_2023_2020_MOESM1_ESM.docx]

**Appendix Table 1: State-wise use of hygienic products and socioeconomic inequality in the use of hygienic products among adolescent girls in India, during NFHS-4 (2015-16) and NFHS-5 (2019-21)**

| **States** | **Use of hygienic products (%)** | | **Change in prevalence**  **(in % points)** | **ECI** | | **Change in ECI** | **P-value** |
| --- | --- | --- | --- | --- | --- | --- | --- |
|  | **NFHS-4** | **NFHS-5** |  | **NFHS-4** | **NFHS-5** |  |  |
| Andhra Pradesh | 65.1 | 69.4 | 4.3 | 0.308 | 0.226 | -0.082 | 0.043 |
| Arunachal Pradesh | 53.9 | 69.2 | 15.3 | 0.397 | 0.274 | -0.123 | <0.001 |
| Assam | 22.2 | 34.8 | 12.5 | 0.315 | 0.307 | -0.008 | 0.719 |
| Bihar | 15.7 | 31.4 | 15.7 | 0.249 | 0.306 | 0.057 | <0.001 |
| Chhattisgarh | 20.4 | 34.1 | 13.7 | 0.324 | 0.289 | -0.035 | 0.080 |
| Goa | 70.9 | 64.6 | -6.3 | 0.289 | 0.157 | -0.131 | 0.222 |
| Gujarat | 49.4 | 47.3 | -2.1 | 0.313 | 0.314 | 0.000 | 0.984 |
| Haryana | 53.9 | 79.1 | 25.2 | 0.325 | 0.201 | -0.124 | <0.001 |
| Himachal Pradesh | 57.4 | 69.6 | 12.2 | 0.280 | 0.234 | -0.045 | 0.275 |
| Jharkhand | 27.5 | 45.0 | 17.5 | 0.377 | 0.343 | -0.035 | 0.087 |
| Karnataka | 45.8 | 57.7 | 12.0 | 0.336 | 0.313 | -0.022 | 0.379 |
| Kerala | 51.3 | 59.4 | 8.1 | 0.299 | 0.277 | -0.021 | 0.611 |
| Madhya Pradesh | 21.5 | 34.8 | 13.2 | 0.388 | 0.456 | 0.068 | <0.001 |
| Maharashtra | 51.4 | 74.3 | 22.9 | 0.341 | 0.303 | -0.038 | 0.077 |
| Manipur | 29.7 | 37.9 | 8.3 | 0.254 | 0.267 | 0.013 | 0.746 |
| Meghalaya | 27.0 | 34.3 | 7.3 | 0.362 | 0.409 | 0.047 | 0.135 |
| Mizoram | 88.1 | 86.7 | -1.4 | 0.190 | 0.258 | 0.069 | 0.020 |
| Nagaland | 43.5 | 44.0 | 0.5 | 0.409 | 0.376 | -0.033 | 0.410 |
| Odisha | 33.3 | 62.7 | 29.4 | 0.382 | 0.347 | -0.035 | 0.108 |
| Punjab | 50.3 | 75.2 | 24.9 | 0.446 | 0.221 | -0.225 | <0.001 |
| Rajasthan | 31.2 | 58.0 | 26.8 | 0.448 | 0.355 | -0.094 | <0.001 |
| Sikkim | 49.2 | 75.4 | 26.2 | 0.216 | 0.198 | -0.018 | 0.773 |
| Tamil Nadu | 86.3 | 87.8 | 1.5 | 0.112 | 0.101 | -0.010 | 0.562 |
| Telangana | 71.8 | 84.2 | 12.4 | 0.280 | 0.122 | -0.158 | <0.001 |
| Tripura | 30.7 | 42.2 | 11.5 | 0.413 | 0.286 | -0.126 | 0.020 |
| Uttar Pradesh | 17.1 | 28.8 | 11.7 | 0.310 | 0.319 | 0.009 | 0.335 |
| Uttarakhand | 42.8 | 62.1 | 19.4 | 0.417 | 0.338 | -0.078 | 0.008 |
| West Bengal | 33.9 | 60.3 | 26.4 | 0.438 | 0.296 | -0.142 | <0.001 |
| India | 36.8 | 50.1 | 13.4 | 0.481 | 0.427 | -0.055 | <0.001 |

Note: NFHS= National Family Health Survey, ECI=Erreygers Concentration Index, all percentages are weighted
